# The Therapeutic Potential of Hemp Seed Oil in D-Galactose-Induced Aging Rat Model Was Determined through the Combined Assessment of ^1^H NMR Metabolomics and 16S rRNA Gene Sequencing

**DOI:** 10.3390/metabo14060304

**Published:** 2024-05-27

**Authors:** Hailong Lu, Lixi Li, Zhongjie Zou, Bin Han, Mengjuan Gong

**Affiliations:** School of Chinese Materia Medica, Guangdong Pharmaceutical University, Guangzhou 510006, China; 2112248129@stu.gdpu.edu.cn (H.L.); 2112248035@stu.gdpu.edu.cn (L.L.); zouzhongjie@gdpu.edu.cn (Z.Z.)

**Keywords:** hemp seed oil, D-gal-induced aging, metabolomics, gut microbiota

## Abstract

Aging is an irreversible process of natural degradation of bodily function. The increase in the aging population, as well as the rise in the incidence of aging-related diseases, poses one of the most pressing global challenges. Hemp seed oil, extracted from the seeds of hemp (*Cannabis sativa* L.), possesses significant nutritional and biological properties attributed to its unique composition of polyunsaturated fatty acids and various antioxidant compounds. However, there is limited knowledge regarding the anti-aging mechanism of hemp seed oil. This study aimed to evaluate the beneficial effects and potential mechanisms of hemp seed oil in a D-galactose (D-gal)-induced aging rat model through a combined analysis of metabolomics and 16S rRNA gene sequencing. Using nuclear magnetic resonance (NMR)-based metabolomics, significant alterations in serum and urine metabolic phenotypes were observed between the D-gal-induced aging rat model and the healthy control group. Eight and thirteen differentially expressed metabolites related to aging were identified in serum and urine, respectively. Treatment with hemp seed oil significantly restored four and ten potential biomarkers in serum and urine, respectively. The proposed pathways primarily included energy metabolism, amino acid metabolism, one-carbon metabolism, and lipid metabolism. Furthermore, 16S rRNA gene sequencing analysis revealed significant changes in the gut microbiota of aged rats. Compared to the model group, the hemp seed oil group exhibited significant alterations in the abundance of 21 bacterial taxa at the genus level. The results indicated that hemp seed oil suppressed the prevalence of pathogenic bacterial genera such as *Streptococcus*, *Rothia*, and *Parabacteroides*. Additionally, it facilitated the proliferation of the genera *Lachnospirace*_NK4B4_group and *Lachnospirace*_UCG_001, while also enhancing the relative abundance of the genus *Butyricoccus*; a producer of short-chain fatty acids (SCFAs). These findings provided new insights into the pathogenesis of aging and further supported the potential utility of hemp seed oil as an anti-aging therapeutic agent.

## 1. Introduction

With the gradual enhancement of living standards, an increasing focus has been directed toward aging. Exploring convenient and effective methods to delay aging has emerged as an intriguing subject. Aging represents an irreversible phenomenon characterizing the gradual and natural deterioration of organismal functions at various levels. This multifaceted phenomenon manifests as compromised internal environmental stability and stress resistance, rendering individuals more susceptible to chronic ailments [1]. Moreover, mounting evidence underscores that judicious dietary adjustments can significantly enhance health and extend lifespan.

With delta-9-tetrahydrocannabinol (THC) content below 0.3%, non-drug cultivars of *Cannabis sativa* L., commonly referred to as “hemp”, have played a significant role in Chinese and European cultures for millennia; serving as a vital source of nutrition, fiber, and therapeutic applications. Particularly, the edible fruits of *C. sativa* L., known as hemp seeds, exhibit remarkable nutritional richness, as they contain abundant lipids, phytosterols, tocopherols, proteins, and functional compounds such as phenolic compounds and bioactive peptides. Techniques such as cold pressing and solvent extraction efficiently extract hemp seed oil [2]. Cold-pressed hemp seed oil boasts an approximate composition of 80% polyunsaturated fatty acids, predominantly comprising vital linoleic acid (18:2 n-6, LA) and α-linolenic acid (18:3 n-3, ALA), aligning harmoniously with the optimal 3:1 ratio recommended by human nutritional guidelines [3]. Furthermore, this oil is notably rich in trace elements, including γ-linolenic acid, a compound uncommonly found in vegetable oils. It also contains significant levels of tocopherols, phytosterols, and other bioactive constituents, showcasing commendable antioxidant and anti-aging properties. Particularly within the Bama region of Guangxi, China, this oil is reputed as “longevity oil” due to its remarkable characteristics. Evidence has emerged supporting the beneficial effects of a dietary combination comprising hemp seed and bitter vegetable (*Sonchus oleraceus*) on health, longevity, and cognitive function [4]. Previous investigations have suggested that hemp seed polyphenols exhibit robust antioxidant activity in vitro and effectively shield human umbilical vein endothelial cells (HUVEC) against oxidative stress damage induced by H_2_O_2_ [5]. However, the precise mechanism behind the anti-aging properties of hemp seed oil remains poorly understood. Therefore, the primary objective of this study is to elucidate the potential mechanism underlying the therapeutic effects of hemp seed oil.

Metabolomics, a well-established field within systems biology, holds significant potential in drug discovery and clinical research. It encompasses various applications, including the identification of novel targets, elucidation of mechanisms of action for new drugs, characterization of safety and efficacy profiles, and the discovery of biomarkers for early disease diagnosis, prognosis, patient stratification, and treatment response monitoring [6]. Numerous metabolomics investigations have revealed substantial variations in metabolite profiles across different age cohorts, spanning diverse categories such as amino acids, carbohydrates, biogenic amines, and lipids. Additionally, these studies have elucidated aging-related metabolic pathways, including amino acid metabolism, lipids, and energy metabolism [7]. Given the absence of prior nuclear magnetic resonance (NMR)-based metabolomics investigations concerning the treatment of rat aging models using hemp seed oil, we conducted an exploratory study to address this research gap.

Recent research has unveiled age-related changes in the composition, diversity, and functional attributes of the gut microbiota in older individuals; this is linked primarily to declines in immune system function, known as immunosenescence, and the presence of low-grade chronic inflammation [8]. Studies in centenarians across multiple countries have highlighted a substantially higher diversity of *Ruminococcaceae* compared to younger individuals [9]. Notably, the gut microbiota of centenarians displays a distinct profile characterized by a reorganization of *Firmicutes* and an enrichment of *Proteobacteria*, including opportunistic pro-inflammatory bacteria referred to as “pathogenic bacteria”. Additionally, *Eubacterium* and related taxa have emerged as significant marker bacteria for longevity, exhibiting over a tenfold increase in abundance among centenarians. These findings suggest that age-related disparities in gut microbiota composition may intricately relate to disease progression and frailty in the elderly [10]. Here, we hypothesize that gut microbiota serve as a target of hemp seed oil in aging intervention.

In the present study, ^1^H NMR-based metabolomics combined with 16s rRNA gene sequencing was used to capture metabolic profile changes and monitor intestinal microbiota changes associated with D-galactose (D-gal)-induced aging in rats and hemp seed oil treatment, which might provide new insights into understanding the potential mechanism of hemp seed oil treatment for aging and its development and utilization.

## 2. Materials and Methods

### 2.1. Chemicals and Reagents

In this study, a commercially available preparation of cold-pressed hemp seed oil (Impression of Life Experience Co., Ltd., Bama, China) was utilized. The fatty acid compositions were reported in our previous study [3]. Deuterium oxide (D_2_O) was procured from Sigma-Aldrich (St. Louis, MO, USA). Water was obtained from a Milli-Q ultra-pure system (Millipore, Bedford, MA, USA).

### 2.2. Animal Experiments

This study was approved by the Committee on the Ethics of Animal Experiments of Guangdong Pharmaceutical University (approval number: gdpuac2022087) and carried out in strict accordance with NIH guidelines for the care and use of laboratory animals.

A total of 18 male SD (Sprague-Dawley) rats, weighing approximately 180 ± 20 g, were procured from Beijing Huafukang Bio-technology Co., Ltd. (Beijing, China). These rats were housed in a specific pathogen-free facility, where environmental conditions including temperature (22 ± 2 °C), humidity (45~55%), and a 12-h light/dark cycle were meticulously controlled. The rats had unrestricted access to water and food throughout the experiment. The experimental model was established and implemented following the methodology described in reference [11]. Following a one-week acclimatization period, rats were randomly assigned to one of three groups (*n* = 6 per group): the control group (0.5% Tween-80 solution), the model group (0.5% Tween-80 solution), and the hemp seed oil group (0.5 g/kg, dissolved in 0.5% aqueous solution of Tween 80, and administrated orally by gavage). The dose level was calculated from the recommended adult human dose of hemp seed (15 g/60 kg or 0.25 g/kg documented in Chinese pharmacopoeia) and the oil content of hemp seed (about 35% of the whole seed reported in the literature [12]) using the following equation: animal dose (g/kg) = human dose (g/kg) × oil content of hemp seed × human *Km*/animal *Km*, where human *Km* (adult) was 37 and animal *Km* (rat) was 6 [13]. Excluding the control group, the remaining two groups received daily subcutaneous injections of D-galactose (120 mg/kg) dissolved in 0.9% saline solution on the back of the neck for a duration of 6 weeks, ensuring uninterrupted administration to establish the experimental model. The control group received an equivalent volume of normal saline injections during the same period. Throughout the experiment, rat body weight was measured every three days, and their physical condition, including fur condition and activity levels, was observed and recorded. Cognitive function tests were conducted on rats following the final administration. At the end of the experiment, 24-h urinary samples and fecal samples acquired between 09:00 am and 17:00 pm were stored at −80 °C for subsequent metabolomics and gut microbiota analysis. An intraperitoneal injection of isoflurane (3 mL/kg) induced anesthesia, followed by blood collection from the abdominal aorta. The blood samples were allowed to clot at room temperature for 60 min, then centrifuged (4000 r/min, 15 min, 4 °C) to isolate serum. Post-blood collection, rats were placed on ice, and their liver and thymus were promptly removed, rinsed with saline, and weighed.

### 2.3. Cognitive Function Assessment

#### 2.3.1. Morris Water Maze

As previously described in studies, cognitive function assessment in rats was conducted using the Morris water maze test [14]. The Morris water maze (MWM) test consisted of 4 training trials of searching for the hidden platform on each of the 5 consecutive days, followed by a spatial probe test on the 6th day, where the hidden platform was located in quadrant III. The study was conducted in a dark circular pool 160 cm in diameter and 50 cm in height, and filled with water maintained at 25 ± 2 °C. During place navigation test, rats were timed from entry into the water until reaching the platform, with this duration recorded as the escape latency, limited to a maximum of 60 s. On the sixth day, during the spatial probe test, the platform was removed. Rats were introduced to the water from quadrant I, opposite to the location of quadrant III, and their movements within the target quadrant, as well as the number of times they crossed the previous platform location within 60 s, were captured and recorded using a camera system.

#### 2.3.2. Open Field Test

An open field test [15] was performed after MWM to evaluate exploration and motor activity. The rats were individually placed in a test box (50 × 50 × 35 cm) for adaptation for 2 min, and then their total distance moved in the box was recorded for 3 min. The test box was cleaned out with 75% ethanol solution and dry paper tissue between all sessions.

### 2.4. Metabolomics Analysis Based on ^1^H NMR Spectroscopy

The serum and urine samples were prepared as per our previously published report [16]. Briefly, serum samples were prepared by adding 50 μL of buffer solution (0.2 M Na_2_HPO_4_ and 0.2 M NaH_2_PO_4_, pH 7.4) and 50 μL of D_2_O to 400 μL of serum. For urine samples, 400 μL of urine was mixed with 200 μL of buffer (0.2 M Na_2_HPO_4_ and 0.2 M NaH_2_PO_4_, pH 7.4) and centrifuged at 4000 r/min for 15 min at 4 °C. The resulting supernatant (500 μL) was transferred to a 5 mm NMR tube, and 50 μL of D_2_O containing 0.05% (*w*/*v*) sodium 3-trimethylsilyl [2,2,3,3-d4] propionate (TSP-d_4_) was added.

NMR spectroscopic data were acquired on a Bruker AVANCE III 500 MHz spectrometer (Bruker Biospin, Rheinstetten, Germany) at a temperature of 298 K. For serum samples, the water-suppressed Carr–Purcell–Meibom–Gill (CPMG) spin-echo pulse sequence (RD-90°-(τ-180°-τ)n-ACQ) with a total spin-echo delay (2 nτ) of 100 ms was used to attenuate broad signals from proteins and lipoproteins. In total, 128 free induction decays (FIDs) were collected into 64 k data points over a spectral width of 10,000 Hz, with a relaxation delay of 3 s and an acquisition time of 3.28 s. For urine samples, all ^1^H NMR spectra were collected using a standard 1D nuclear overhauser enhancement spectroscopy (NOESY)-presaturation pulse sequence. In total, 128 free induction decays (FIDs) were collected into 64 k data points. Spectra were acquired with a spectral width of 10,000 Hz and an acquisition time of 3.28 s. Relaxation delay was set at 3 s. Spectral assignments were performed using Human Metabolome Database, Chenomx NMR Suite (Chenomx Inc., Edmonton, AB, Canada), and previously published data [17].

The acquired spectra were processed with a 0.3 Hz line-broadening factor prior to Fourier transformation, and then phase and baseline distortions were manually adjusted using MestReNova 12.0 software (Mestrelab Research S.L., Santiago de Compostela, Spain). Serum and urine samples were calibrated with reference to the methyl resonance of lactate (δ 1.33) and TSP (δ 0.0), respectively. The spectral region from δ 0.5 to 9.5 was divided into equal intervals of δ 0.01 for integration. To eliminate the interference of the water peak, the regions δ 4.68–5.22 and δ 4.49–5.99 were set as integration segments with an integral value of 0 for serum and urine samples, respectively. Subsequently, all remaining spectral integration regions were normalized to the total sum of the spectrum and multiplied by 10,000 before pattern recognition analysis. The Pareto-scaled NMR data were subjected to multivariate statistical analysis using commonly employed tools such as principal component analysis (PCA) and orthogonal partial least-squares discriminant analysis (OPLS-DA) using SIMCA 12.0 software (Umetrics, Umea, Sweden).

In order to ensure robustness and prevent overfitting, a sevenfold cross-validation strategy was employed, and statistical parameters including R^2^Y (representing the goodness of fit) and Q^2^ (representing the predictive ability) were calculated. To further validate the models, permutation tests were performed to assess their quality. Identification of potential biomarkers was carried out based on the following criteria: metabolites with VIP (variable importance in the projection) values ≥ 1.0 (obtained from the OPLS-DA models) and statistical significance with *p* < 0.05 determined through Student’s *t*-test (equal variances assumed) or Mann–Whitney *U*-test (equal variances not assumed).

### 2.5. 16S rRNA-Based Microbial Community Analysis

Total genomic DNA of the gut microbiota was extracted from fecal samples using the PowerSoil DNA Isolation Kit (MO BIO Laboratories, Inc., Carlsbad, CA, USA) with bead-beating, ensuring efficient lysis of microbial cells. Subsequently, the V3-V4 variable regions of the bacterial 16S rRNA gene were specifically amplified using universal primers (338F 5′-ACTCCTACGGGAGGCAGCA-3′ and 806R 5′-GGACTACHVGGGTWTCTAAT-3′) that incorporated unique barcodes for each sample, enabling sample multiplexing. Amplicon sequencing was performed using the state-of-the-art Illumina Novaseq 6000 platform. The generated sequencing data were subjected to bioinformatics analysis, including quality control, read merging, taxonomic classification, and diversity analysis; which were conducted by BMKCloud (https://www.biocloud.net) (accessed on 17 June 2023).

Quantitative Insights Into Microbial Ecology (QIIME, v1.8.0) was employed for data analysis in the field of microbial ecology. The raw reads underwent demultiplexing and quality filtering using programs such as FLASH, Trimmomatic, and UCHIME. Sequence clustering of the samples was performed using USEARCH v10.0 at a 97% similarity threshold to generate operational taxonomic units (OTUs), representing clusters of similar sequences. Taxonomic annotation of the OTUs was carried out using the SILVA (http://www.arb-silva.de) (accessed on 14 June 2023) and UNITE (unite.ut.ee/index.php) (accessed on 15 June 2023) databases, along with the RDP Classifier v2.2 software, with a confidence threshold set at 0.8.

The alpha diversity indices—including Chao1, Simpson, and Shannon indices—were indicative of the species richness and diversity within individual samples. Beta diversity analysis was employed to assess the extent to which different samples exhibited similarity in terms of species diversity. QIIME software was used to calculate the distance between samples and, subsequently, Beta diversity analysis was conducted to assess the similarity of different samples in terms of species diversity. The unweighted UniFrac method was selected to obtain the distance between samples. Alpha rarefaction and principal coordinates analysis (PCoA) were used to assess diversities in the gut microbial communities. A PCoA analysis map was drawn to show the microbial beta diversity. The analysis employed the linear discriminant analysis (LDA) effect size (LEfSe) method (http://huttenhower.sph.harvard.edu/lefse/) (accessed on 17 June 2023)—a statistical approach with biological relevance—with the capability to identify biomarkers exhibiting statistically significant differences between different groups, to discern microbial taxa exhibiting significant differences [18].

### 2.6. Statistical Analysis

All data were reported as mean ± standard deviation (SD). Statistical differences were assessed using one-way analysis of variance (ANOVA) followed by Tukey’s post hoc test, performed with SPSS 17.0 software (SPSS Inc., Chicago, IL, USA). A significance level of *p* < 0.05 was considered statistically significant. Pearson correlation analysis was utilized to evaluate the relationship between gut microbes and metabolites in serum and urine. Pearson’s correlation co-efficient was calculated and visualized graphically using GraphPad Prism 8.0 software (GraphPad Software, Inc., San Diego, CA, USA).

## 3. Results

### 3.1. Hemp Seed Oil Ameliorates D-Gal-Induced Aging in Rats

**Figure 1:**
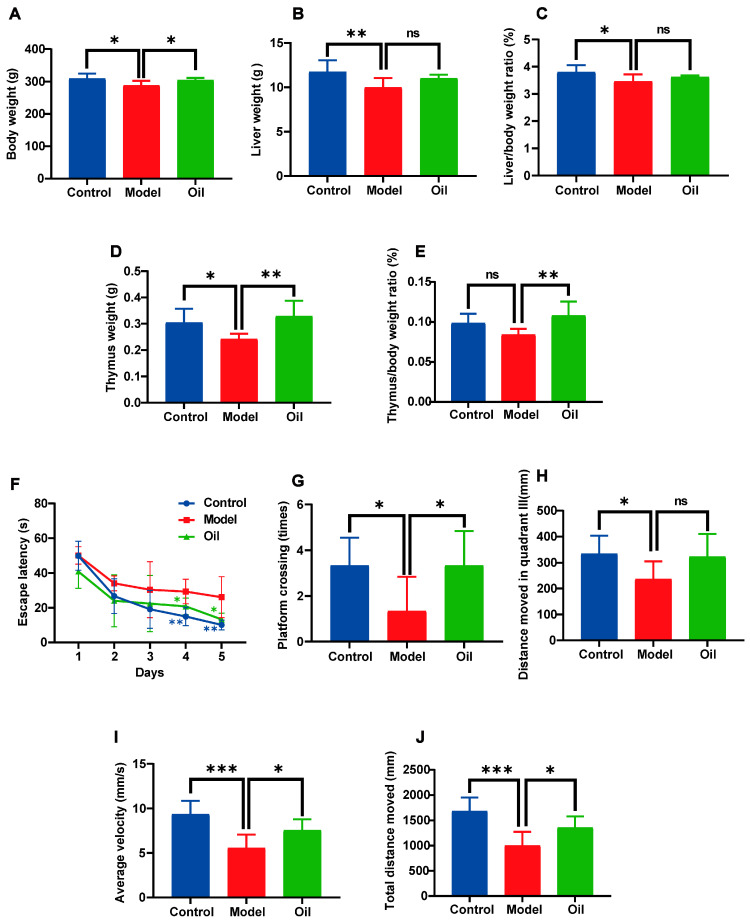
Characteristics of D-galactose-induced aging in rats with or without hemp seed oil treatment for 7 weeks. (**A**) Body weight. (**B**) Liver weight. (**C**) Liver/body weight ratio. (**D**) Thymus weight. (**E**) Thymus/body weight ratio. (**F**) Escape latency. (**G**) The number of platform crossings in 60 s. (**H**) Distance moved in quadrant III. (**I**) Average velocity. (**J**) Total distance moved. Values are presented as mean ± standard deviation (SD), *n* = 6 per group. Differences were assessed by one-way analysis of variance (ANOVA) followed by Tukey’s post hoc test. Compared with the model group, * *p* < 0.05, ** *p* < 0.01, *** *p* < 0.001, ns: not significant.

Following subcutaneous injections of D-galactose solution for 6 weeks, rats exhibit significant reductions in body weight, liver weight, liver/body weight ratio, and thymus weight. Although there is a decrease in the thymus/body weight, it is not statistically significant. Treatment with hemp seed oil results in an increase in the aforementioned parameters, with significant elevations observed in body weight, thymus weight, and thymus/body weight ratio (Figure 1A–E).

The results of spatial navigation training reveal that, during the training period, D-gal-induced aged rats exhibit longer escape latencies to reach the platform compared to the control group. However, hemp seed oil significantly reduces the escape latency (Figure 1F). In terms of spatial probe test, the number of platform crossings and the distance moved in quadrant III notably decrease in the aging model rats (Figure 1G,H). Hemp seed oil increases the distance moved in quadrant III and significantly enhances the number of platform crossings. The model group of rats exhibits a significant decrease in both total distance moved and average velocity compared to the control group, as presented in the results of the open field test. In contrast, rats treated with hemp seed oil show a significant increase in both total distance moved and average velocity (Figure 1I,J). Our results imply that hemp seed oil possesses potential beneficial properties that mitigate the effects of D-gal-induced aging in rats.

### 3.2. Hemp Seed Oil Treatment Alleviates Metabolic Disorders in Rats with D-Gal-Induced Aging

**Figure 2:**
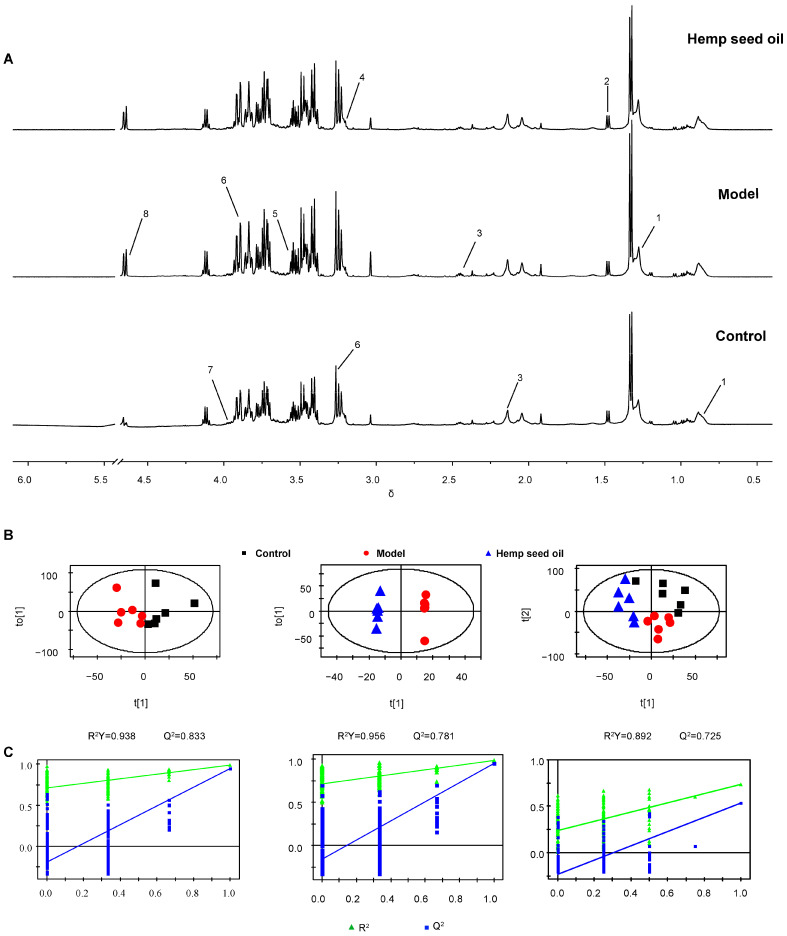
Serum metabonomic analysis. (**A**) Representative ^1^H NMR spectra of rat serum. Key: 1, VLDL/LDL; 2, Alanine; 3, Glutamine; 4, Choline; 5, Glycine; 6, Betaine; 7, Phenylalanine; 8, β−glucose. (**B**) OPLS−DA score plots. (**C**) The corresponding validation plots based on 200 times permutation tests. The OPLS−DA models were valid and not over-fitted as the R^2^ and Q^2^ values derived from the permuted data were lower than the original ones and all the blue regression lines of the Q^2^−points intersected the vertical axis below zero.

**Figure 3:**
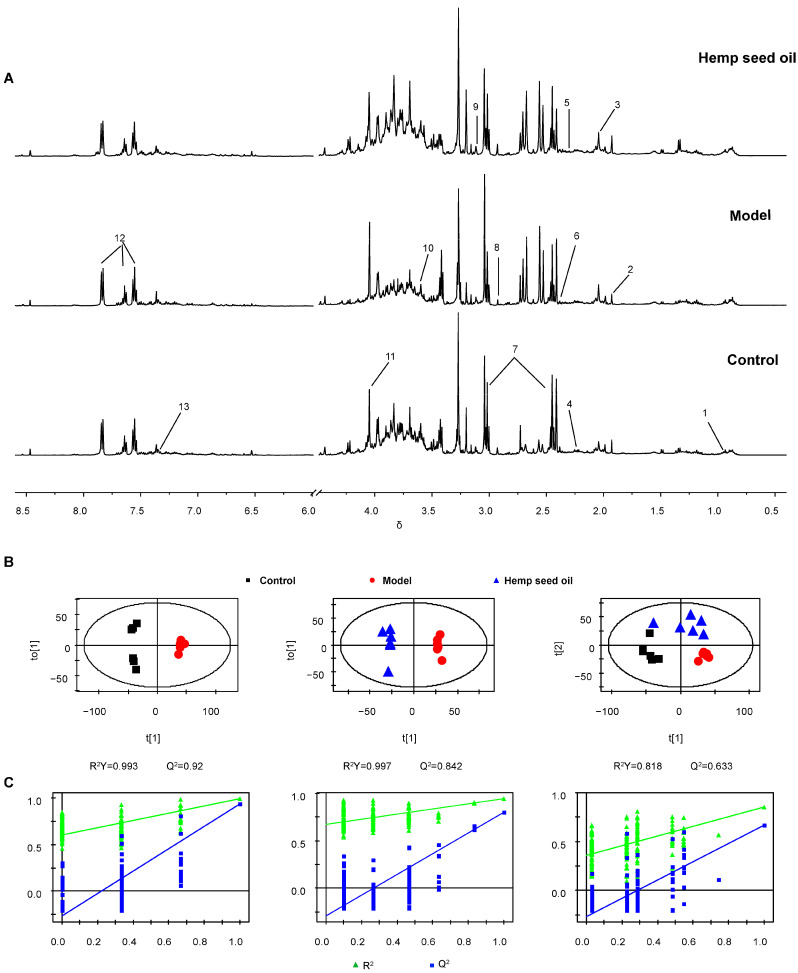
Urinary metabolomic analysis. (**A**) Representative ^1^H NMR spectra of rat urine. Key: 1, Isoleucine; 2, Acetate; 3, *N*−Acetyl glycoprotein; 4, Acetone; 5, Acetoacetate; 6, Pyruvate; 7, α−Ketoglutarate; 8, Dimethylglycine; 9, Cysteine; 10, Sarcosine; 11, Creatinine; 12, Hippurate; 13, Tryptophan. (**B**) OPLS−DA score plots. (**C**) The corresponding validation plots based on 200 times permutation tests. The OPLS−DA models were valid and not over−fitted, as the R^2^ and Q^2^ values derived from the permuted data were lower than the original ones and all the blue regression lines of the Q^2^−points intersected the vertical axis below zero.

The ^1^H-NMR spectra of rat serum and urine are shown in Figure 2A and Figure 3A. Major endogenous metabolites identified by analysis are assigned and labeled in the spectra. In order to study metabolic disorder induced by aging in rats and to reveal the effect of intervention with hemp seed oil, OPLS-DA score plots are constructed based on normalized NMR spectral data obtained from rat serum and urine samples. A clear separation of serum and urine metabolic phenotypes is observed in control and model rats (Figure 2B and Figure 3B). The results show that the metabolism of aging rats induced by D-gal has obvious changes. In addition, the OPLS-DA score plot shows complete differentiation of serum and urine metabolic profiles between the model group and the hemp seed oil group, and shows a trend of recovery to the healthy control group in the hemp seed oil group, which may be due to the therapeutic effect of hemp seed oil against aging (Figure 2B and Figure 3B). Both the model parameters of R^2^Y and Q^2^ and the results of permutation tests illustrate the validity of the OPLS-DA models (Figure 2C and Figure 3C).

**Figure 4:**
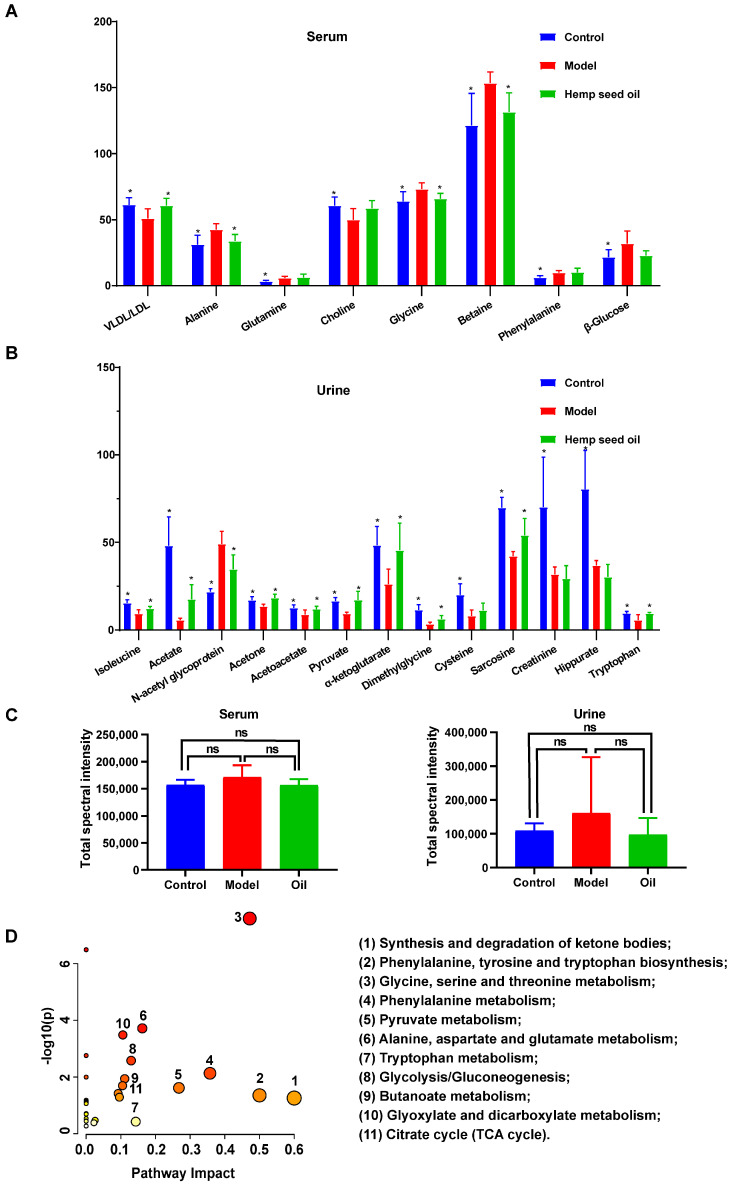
Differential metabolites associated with aging. (**A**,**B**) Relative levels of differential metabolites related to aging in serum and urine of rats. Error bars indicated the mean ± SD (*n* = 6). * represents *p* < 0.05 compared with the model group. (**C**) Total spectral intensity. Values are presented as mean ± standard deviation (SD), *n* = 6 per group. ns: not significant. (**D**) Summary of pathway analysis with MetaboAnalyst. (1) Synthesis and degradation of ketone bodies; (2) Phenylalanine, tyrosine and tryptophan biosynthesis; (3) Glycine, serine and threonine metabolism; (4) Phenylalanine metabolism; (5) Pyruvate metabolism; (6) Alanine, aspartate and glutamate metabolism; (7) Tryptophan metabolism; (8) Glycolysis/Gluconeogenesis; (9) Butanoate metabolism; (10) Glyoxylate and dicarboxylate metabolism; (11) Citrate cycle (TCA cycle).

Based on the VIP (Variable Importance in Projection) values (VIP ≥ 1) and the *p* values obtained from univariate statistical analysis (*p* < 0.05), we identify eight significantly altered endogenous metabolites associated with aging in serum, as well as 13 in urine, which potentially serve as biomarkers (Figure 4A,B, and Table 1). Compared to the control group, the serum metabolic profile of rats in the aging model exhibits notable changes: increased levels of alanine, glutamine, glycine, betaine, phenylalanine, and β-glucose; alongside decreased VLDL/LDL and choline levels. Simultaneously, the urine metabolic characteristics of the model group differ significantly from the control: elevated N-acetyl glycoprotein levels and notable reductions in isoleucine, acetate, acetone, acetoacetate, pyruvate, α-ketoglutarate, dimethylglycine, cysteine, sarcosine, creatinine, hippurate, and tryptophan levels. Furthermore, hemp seed oil is found to significantly reverse the levels of all potential aging-associated biomarkers in rats, except for glutamine, phenylalanine, β-glucose, and choline in serum, as well as creatinine, hippurate, and cysteine in urine (Figure 4A,B, and Table 1).

The total spectral intensity serves as a quantitative measure of the cumulative metabolite content within samples, facilitating the observation of broad-scale shifts in metabolic profiles and inter-sample congruence. By comparing the total spectral intensity of different sample groups, the results indicate that there are no statistically significant differences in total spectral intensity between groups; demonstrating the consistency of overall metabolite levels between different samples (Figure 4C).

**Figure 5:**
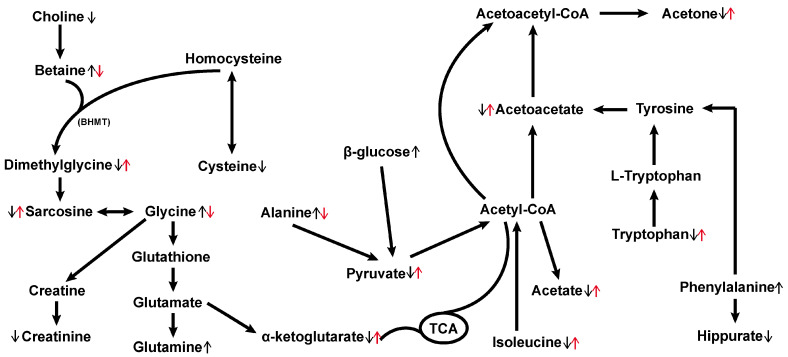
Schematic representation of the changed metabolic pathways in aged rats. ↑ represents significant up-regulations of metabolites in the model group compared with the control group, whereas ↓ indicates down-regulations. ↑ (red) represents significant up-regulations of metabolites in the hemp seed oil group compared with the model group, whereas ↓ (red) indicates down-regulations. BHMT: betaine-homocysteine S-methyltransferase, TCA: tricarboxylic acid cycle.

The metabolic pathway analysis conducted using MetaboAnalyst (www.metaboanalyst.ca/MetaboAnalyst) (accessed on 26 April 2023) aimed to explore the pathways associated with D-gal-induced aging in rats. Based on the impact value greater than 0.1, the disturbed metabolic pathways (Figure 4D and Figure 5) primarily encompassed the synthesis and degradation of ketone bodies, phenylalanine, tyrosine and tryptophan biosynthesis, glycine, serine, and threonine metabolism, phenylalanine metabolism, pyruvate metabolism, alanine, aspartate, and glutamate metabolism, tryptophan metabolism, glycolysis/gluconeogenesis, butanoate metabolism, glyoxylate and dicarboxylate metabolism, and the Citrate cycle (TCA cycle); these pathways exhibit promising potential as targetable anti-aging pathways for hemp seed oil.

### 3.3. Hemp Seed Oil Treatment Modulates Gut Microbiota Composition in D-Gal-Induced Aged Rats

**Figure 6:**
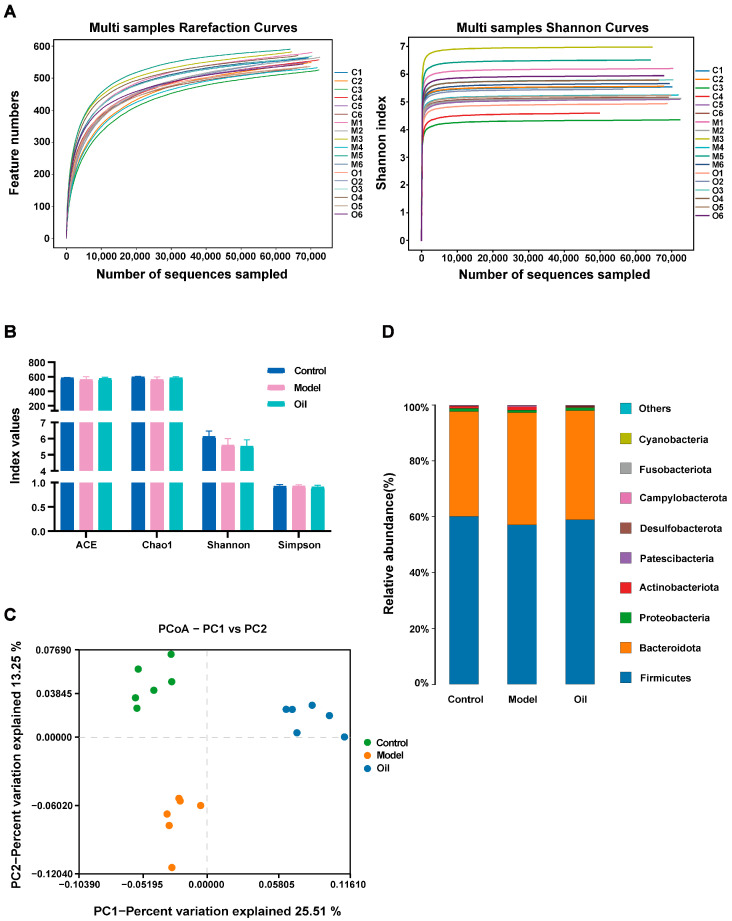
16S rRNA gene sequencing analysis of fecal microbiome. (**A**) Rarefaction and Shannon curves. (**B**) Alpha diversity indices. (**C**) PCoA score plot. (**D**) Relative abundance of gut microbiota at the phylum level.

**Figure 7:**
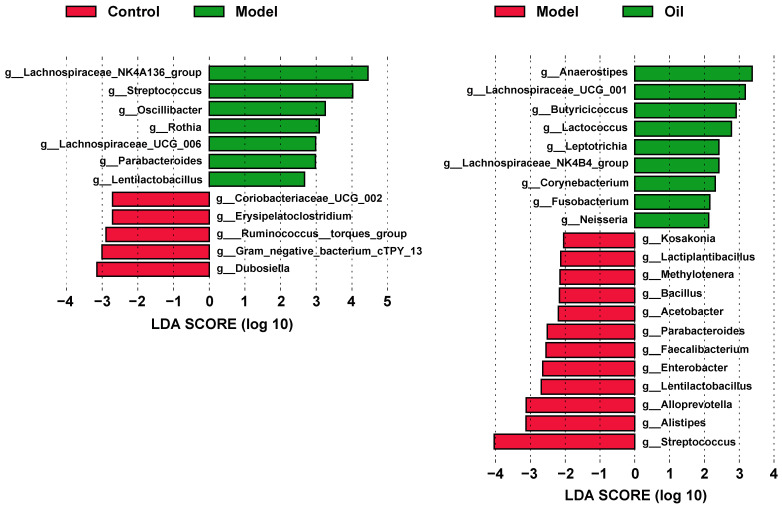
Histogram of the LEfSe LDA scores computed for differential taxonomic clades between paired groups. Only taxa with an LDA value > 2 are shown. The color (red or green) indicates the enrichment of the taxa within the corresponding groups. The letters g represent genus.

In 16S rRNA gene sequencing, a total of 1,442,299 pairs of reads are generated. After undergoing paired-end reads splicing, quality control filtering, and chimera removal, a total of 1,434,216 valid sequences are obtained, with an average of 79,678 valid sequences per sample. Subsequently, clustering is conducted at a similarity level of 97.0%, resulting in the identification of 645 operational taxonomic units (OTUs). The rarefaction and Shannon curves demonstrate that the sequencing depth adequately covers all species within the sample, encompassing the vast majority of microbial species information (Figure 6A). Regarding alpha diversity—as assessed by the ACE, Chao1, Shannon, and Simpson indices (Figure 6B)—no significant differences are observed among the three groups. However, the PCoA score plot demonstrates notable alterations in the overall structure and composition of the intestinal microbiota in D-gal-induced aged rats, which are ameliorated by the administration of hemp seed oil (Figure 6C). Taxonomic analysis conducted at the phylum level reveals that Firmicutes and Bacteroidota constituted the prevailing components of the fecal microbial communities across all groups (Figure 6D). To identify differential bacterial taxa at genus levels, we employ the Linear discriminant analysis Effect Size (LEfSe) method. Significantly altered taxa with Linear Discriminant Analysis (LDA) scores exceeding 2.0 are selected for further analysis and interpretation. The analysis of microbial differences between the control group and the model group reveals alterations in 12 genera at the genus level within the model group compared to the control group (Figure 7). Furthermore, the hemp seed oil group exhibits changes in 21 genera at the genus level as compared to the model group. Intriguingly, following treatment with hemp seed oil, a significant restoration in the abundance of three microbial taxa is observed, including g_*Streptococcus*, g_*Parabacteroides*, g_*Lentilactobacillus*.

### 3.4. Correlation of Gut Microbiota and Metabolic Phenotype in D-Gal-Induced Aged Rats

**Figure 8:**
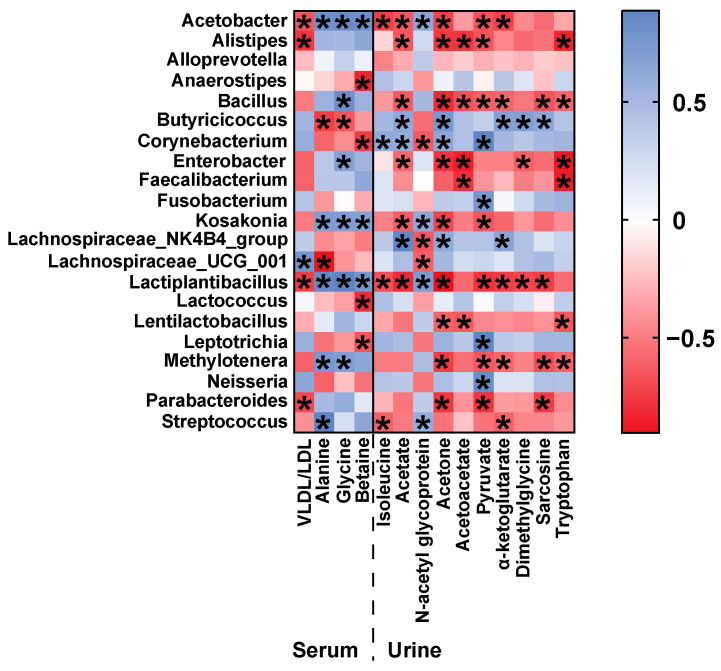
Heat map of the correlation between model group and hemp seed oil group on serum and urine differential metabolites and differential gut microbiota. The colors range from red (negative correlation) to blue (positive correlation). * *p* < 0.05 shows a significant correlation.

To investigate potential functional relationships between the gut microbiome and metabolome, we conduct a Pearson’s correlation analysis between taxonomic genus-level differential microbiota and serum and urine metabolites potential biomarkers, modulated by hemp seed oil (Figure 8). *Lachnospirace*_NK4B4_group, *Lachnospirace*_UCG_001, and *Butyricicoccus* are all bacteria capable of producing short-chain fatty acids. In the hemp seed oil group, *Butyricicoccus* shows a strong positive correlation with urinary acetate levels, whereas *Lachnospirace*_NK4_group and *Lachnospirace*_UCG_001 show a negative correlation with urinary N-acetyl glycoprotein levels.

## 4. Discussion

The global phenomenon of population aging remains a focal concern, rooted fundamentally in the process of aging itself. However, aging represents an irreversible aspect of life, prompting scholars worldwide to actively probe its underlying mechanisms while concentrating on potential pharmaceutical interventions capable of retarding this natural progression. In contemporary studies, there has been a burgeoning interest in exploring methodologies to impede the aging process and extend the period of healthy living through dietary modifications or pharmaceutical interventions [19,20].

Hemp seed oil exhibits diverse biological activities. Prior studies have demonstrated its potential in enhancing antioxidant capacity, counteracting aging processes, and improving cognitive function [3,4,5]. In the present study, our findings offer valuable insights and a comprehensive understanding of the D-galactose-induced aging model, elucidating the role of hemp seed oil in modulating metabolic networks and gut microbiota. Currently, commonly used animal models for aging research include: D-galactose-induced aging models, natural aging models, rapid aging mouse models, ozone damage aging models, and thymectomy aging models, among others. Studies have demonstrated that D-galactose is capable of effectively inducing and accelerating the aging process. The mechanism by which D-galactose induces aging in experimental animals through subcutaneous or intraperitoneal injection involves the excessive accumulation of galactitol in vivo. This accumulation leads to metabolic disorders, cell swelling, and the generation of free radicals; ultimately inducing cell apoptosis and resulting in the decline of multiple organ functions [21]. This model offers advantages such as cost-effectiveness, simplicity, ease of operation, and reliable outcomes; making it widely utilized by both domestic and international researchers [22]. In this study, we established an aging model by administering daily subcutaneous injections of D-galactose solution at a dosage of 120 mg/kg at the nape of the neck. The results of classic physiological indicators and cognitive function assessments confirmed the successful establishment of the aging model, consistent with previous findings [23]. The macroscopic signs, physiological indices, and behavioral indices of aging rats primarily included body weight, coat condition, organ index, mental state, and activity. Experimental findings indicated that the aging model rats experienced a significant decrease in body weight, as well as hair loss and cognitive decline. These observations could be interpreted as characteristic manifestations of the aging process in rats. The cognitive function changes associated with aging are commonly evaluated using the Morris water maze and open field test. The results demonstrated a notable decrease in the capacity of rats within the model group to locate the platform, explore novel environments, and engage in autonomous activities. However, administration of hemp seed oil demonstrated effective improvements in both cognitive function changes and the aforementioned classic physiological indicators in these rats. The omega-3 fatty acids found in hemp seed oil has the potential to enhance the health and functionality of nerve cells, thereby promoting cognitive function. Furthermore, the presence of antioxidants and anti-inflammatory compounds in hemp seed oil might have contributed to a reduction in neuroinflammation and oxidative stress, ultimately safeguarding the nervous system and enhancing cognitive function. These mechanisms were likely to have interacted synergistically, leading to an overall improvement in cognitive function. These findings suggested that hemp seed oil had the potential to mitigate aging-related symptoms, such as cognitive decline, and improve the overall aging process.

Energy metabolism undergoes notable changes during normal development and aging across a wide range of organisms, resulting in imbalances that impact cellular and tissue homeostasis. Extensive studies have implicated various metabolic pathways—including the tricarboxylic acid (TCA) cycle, pyruvate metabolism, glyoxylate and dicarboxylic acid metabolism, and glycolysis/gluconeogenesis—in energy metabolism. The TCA cycle represents the most efficient means of oxidizing and decomposing sugars and other substrates to generate energy. Within this cycle, α-ketoglutarate holds a significant position as a key metabolic intermediate, influencing the overall rate of TCA within the organism. In adddition to its role as an antioxidant, α-ketoglutarate also modulates nitrogen and ammonia balance, while exerting effects on epigenetic and immune regulation. The pleiotropic functions exhibited by α-ketoglutarate hint at its potential for extending the healthy lifespan of humans [24]. Notably, studies have revealed that α-ketoglutarate can inhibit ATP synthase, resulting in reduced ATP content and the inhibition of the TOR pathway, thereby prolonging the lifespan of adult Caenorhabditis elegans and delaying various signs of nematode aging [25]. Pyruvate, the final metabolite of typical cytoplasmic glycolysis, undergoes interconversion with acetyl-CoA and enters the tricarboxylic acid cycle, allowing for the interchange of the three major nutrients in the body. By enhancing the NAD/NADH ratio, pyruvate has the potential to rectify glucose metabolism disorders, prevent multi-organ dysfunction, and improve aging symptoms; and its levels have been observed to decrease in the aging mouse brain [25,26,27]. Acetate, primarily derived from glucose via the glycolysis pathway leading to pyruvate, is subsequently reduced to acetyl-CoA or decarboxylated to CO_2_ through the reductive acetyl-CoA pathway [28]. Within this study, the levels of α-ketoglutarate, pyruvate, and acetate were found to be significantly decreased in aging model rats induced by D-galactose. However, following intervention with hemp seed oil, these metabolite levels were significantly increased. Thus, these findings implied that hemp seed oil exhibited the potential to alleviate the age-related decline observed in the content of intermediate metabolites within the TCA cycle, thereby exerting a discernible influence on energy metabolism.

In comparison to the control group, the model group exhibited notably elevated serum levels of alanine, glutamine, glycine, and phenylalanine; alongside markedly reduced levels of urinary isoleucine, cysteine, and tryptophan. These findings suggested that D-gal-induced senescence might have been related to amino acid metabolism disorder in rats. Prior studies have indicated that glutamine serves as a precursor for the essential antioxidant glutathione, and alterations in its levels can impact lipid peroxidation damage [29]. Furthermore, glutamine plays a crucial role in the TCA cycle, facilitating the export of citrate from mitochondria to the cytoplasm and generating acetyl-CoA, which promotes lipid metabolism. Compared to the control group, the model group rats displayed marked increases in serum levels of alanine and β-glucose, suggesting a potential disruption in the glucose-alanine cycle in the aging rat model. Alanine plays a pivotal role in the glucose-alanine cycle by regulating gluconeogenesis and glycolysis through the inhibition of pyruvate kinase [30]. Moreover, alanine can be converted into pyruvate for oxidative energy supply. Both glycine and alanine serve as precursor amino acids for gluconeogenesis. Glycine, which is derived from glucose, participates in energy metabolism and regulates the production of acetyl-CoA in the body. In the D-gal-induced aging model, aerobic metabolism was reduced, resulting in inadequate energy supply. Consequently, protein breakdown occurred to generate gluconeogenic precursor amino acids, thus increasing gluconeogenesis to meet energy demands. Cysteine, an inherent amino acid recognized for its antioxidant properties, contributes to the synthesis of glutathione in conjunction with glutamic acid and glycine within living organisms [31]. Hemp seed oil had the potential to elevate cysteine levels, potentially mitigating the effects of aging-induced oxidative stress, declining immunity, and inflammation. Previous studies have demonstrated that protein restriction and its benefits in terms of metabolism and aging involve a reduction in the consumption of three branched-chain amino acids (BCAAs): leucine, isoleucine, and valine. Branched-chain amino acids are considered key regulators of healthy metabolism and longevity in both rodents and humans [32]. Isoleucine also influences energy metabolism, as it serves as an essential ketogenic and glucogenic amino acid. It can be converted into succinyl-CoA or acetyl-CoA for oxidation in the TCA cycle. Remarkably, intervention with hemp seed oil significantly restored the isoleucine metabolism abnormality, suggesting its potential to improve the metabolic profile. In this study, the model group exhibited decreased levels of tryptophan, leading to corresponding reductions in its downstream products. Tryptophan, an essential amino acid in humans, undergoes metabolism via the kynurenine pathway. Within this pathway, tryptophan is metabolized into kynurenine, which is further converted into kynurenic acid, anthranilic acid, and NAD+. These metabolites exerted influence over cell aging and susceptibility to oxidative stress. Research has demonstrated that kynurenine can bind to the aromatic hydrocarbon receptor (AhR), thereby stimulating AhR expression [33]. However, high expression of AhR can trigger inflammatory responses, whereas a deficiency in AhR has been shown to improve vascular function and extend the healthy lifespan of model organisms such as C. elegans [34]. Following treatment with hemp seed oil, adjustments in tryptophan levels were observed, indicating its potential to modulate this metabolic pathway.

Disturbances in one-carbon metabolism and elevated levels of homocysteine have been previously associated with the development of Alzheimer’s disease (AD) and Parkinson’s disease (PD)-related dementia [35]. In one-carbon metabolism, betaine, a methyl donor derived from choline oxidation, undergoes methyl transfer to homocysteine via betaine-homocysteine S-methyltransferase (BHMT), leading to the production of dimethylglycine, which contains two methyl groups. Dimethylglycine can then be further degraded to sarcosine and finally to glycine. Additionally, homocysteine is an intermediate product of methionine and cysteine metabolism, which can also be catabolized to cysteine through sulfur transfer pathway. Research has indicated that choline exhibits anti-aging properties, and the resultant betaine from its oxidation triggers NF-κB activation. This maintains the sulfhydryl status during the aging process, thereby modulating COX-2 and TNF-α [36]. NF-κB activity corresponds to the upregulation of NIK/IKK and MAPKs induced by oxidative stress. Hence, betaine, formed through choline’s irreversible oxidation in vivo, may serve as an effective agent in regulating inflammation and aging [37]. Our metabolomics investigation revealed a substantial decrease in serum choline levels, a notable increase in betaine levels, and significant reductions in urine dimethylglycine, cysteine, and sarcosine levels in aging model rats. These alterations indicated a disrupted one-carbon metabolism in the aging model rats. However, hemp seed oil might have potentially reversed these changes by enhancing methyl donors, diminishing oxidative stress, and mitigating inflammatory reactions.

Previous studies have indicated a close association between lipid metabolism disruption and the aging process [38]. In comparison to the control group, the level of N-acetyl glycoprotein was increased in the model group. N-acetyl glycoprotein is an inflammatory mediator that plays a crucial role in maintaining immune function. The elevated levels of N-acetyl glycoprotein indicated the presence of inflammatory reactions and increased protein degradation during aging. The decrease in serum VLDL/LDL levels in the model group might have been attributed to reduced fat synthesis, which supplied energy to the body. This reduction indicated an energy-deficient state, which was restored following drug intervention, suggesting that hemp seed oil had a regulatory effect on lipoproteins, thereby modulating lipid metabolism.

The advent of next-generation sequencing technologies has enabled meticulous examination of the gut microbiome in aging individuals, encompassing both patients and animal models. These investigations have unveiled the manifestation of gut dysbiosis, underscoring the conceivable implication of the gut microbiome in the pathogenesis of aging. Such findings shed light on the potential role of the gut microbiome in the intricate process of aging [8,9,10]. Compared to the control group, the model group exhibited a significant increase in the relative abundance of *Streptococcus*, *Rothia* and *Parabacteroides* at the genus level. *Streptococcus* can cause various infections in the human body, ranging from mild conditions such as pharyngitis and impetigo, to more severe ailments like necrotizing fasciitis and streptococcal toxic shock syndrome. Repeated streptococcal infections may also lead to autoimmune diseases [39]. *Rothia* is classified as a Gram-positive bacterium, which can potentially act as a pathogen in individuals with both compromised and normal immune functions. It has been associated with various infections, particularly affecting the digestive system, by activating human macrophages and lymphocytes. These infections include abdominal infections, periodontitis, and gastrointestinal mucositis [40]. Contrary to prior studies [41], our research uncovered a significant increase in *Parabacteroides* abundance within the aging rat model group, whereas post-administration of hemp seed oil notably decreased its levels. Additionally, research has identified a marked increase in the abundance of microorganisms linked to inflammation, such as *Parabacteroides*, within the gut microbiota of individuals suffering from severe sepsis [39]. Notably, our administration of hemp seed oil resulted in a substantial reduction in the abundance of *Parabacteroides*. Previous studies have demonstrated a decrease in the abundance of Firmicutes in elderly individuals [8]. In our study, a decrease in the abundance of *Ruminococcus* and *Dubosiella*—both members of the Firmicutes phylum and possessing the ability to produce short-chain fatty acids (SCFAs)—was observed in the model group when compared to the control group. SCFAs—such as acetate, propionate, and butyrate—are the primary products of microbial fermentation of carbohydrates in the intestinal tract. They function as intracellular signaling factors, binding to SCFA receptors, and play a crucial role in maintaining metabolic homeostasis. SCFAs exhibit various beneficial functions for host health, including influencing the intestinal nervous system to preserve intestinal barrier integrity, thereby preventing the overgrowth of potential pathogenic bacteria in the gut and promoting systemic anti-inflammatory properties. Additionally, SCFAs are involved in normal microglial development and may impact central nervous system (CNS) epigenesis. The observed increase in pathogenic bacteria, such as *Streptococcus*, *Rothia,* and *Parabacteroides* in the aging rat model suggested that aging might have triggered inflammatory reactions and pathogen infections, leading to reduced immune function and decreased levels of intestinal SCFAs, resulting in disrupted intestinal metabolism. Hemp seed oil demonstrated significant effectiveness in reducing the levels of *Streptococcus* and *Parabacteroides*, indicating its potential to alleviate inflammatory and toxic reactions in aging organisms. In comparison to the model group, the hemp seed oil group exhibited a significant increase in the relative abundance of *Butyricicoccus* and *Lactococcus*. *Butyricicoccus* is a representative bacterium among SCFA-producing microbes, which is specifically known for its significant production of butyrate. Butyrate, which regulates epigenetic processes by inhibiting histone deacetylase activity, is considered to play a crucial role in aging and age-related diseases [42]. Furthermore, research has shown that blueberry mulberry extract (BME), which is rich in polyphenols, possesses antioxidant properties and significantly enhances the relative abundance of *Lactococcus* in the gut. This effect may contribute to alleviating cognitive impairment associated with aging and suppressing inflammation in both the brain and gut [43]. Recent documentation suggests that the microbiota–gut–brain axis not only impacts cognition and psychiatric symptoms but also contributes to the onset of neurodegenerative diseases such as Alzheimer’s disease (AD), Parkinson’s disease (PD), and multiple sclerosis (MS) [44]. In this study, hemp seed oil was administered to aging rats to improve gut microbiota imbalance, maintain brain homeostasis, regulate inflammation, and potentially improve the aging process based on the theory of the brain–gut axis.

In conclusion, this study demonstrated that the administration of hemp seed oil resulted in a reversal of 4 and 10 differential metabolites related to aging in the serum and urine of the model rats, respectively. These findings suggested that hemp seed oil exerted anti-aging effects by partially restoring the balance of disrupted metabolic pathways, including energy metabolism, amino acid metabolism, one-carbon metabolism, and lipid metabolism. Additionally, hemp seed oil was observed to induce alterations in the overall structure and composition of the intestinal microbiota in D-gal-induced aged rats. Compared to the model group, the hemp seed oil group exhibited significant alterations in the abundance of 21 bacterial taxa at the genus level. Specifically, hemp seed oil was able to promote the production of acetate, a short-chain fatty acid (SCFA), by the genus *Butyricoccus*, and it also promoted the abundance of the genera *Lachnospirace*_NK4B4_group and *Lachnospirace*_UCG_001. Furthermore, hemp seed oil was observed to inhibit the relative abundance of pathogenic bacterial genera such as *Streptococcus*, *Rothia*, and *Parabacteroides*. These findings proposed that modulation of the gut microbiota represented one of the mechanisms through which hemp seed oil improved aging. These results provided novel insights into the pathogenesis of aging and further supported the potential therapeutic use of hemp seed oil as an anti-aging intervention.

While potential biomarkers associated with aging were identified and the effects of intervention with hemp seed oil were clearly observed, there were limitations in the current study. Firstly, the underlying mechanisms of the observed changes in metabolic phenotype warrant further investigation, with a goal to verify potential biomarkers in human afflicted with aging and test new medications for their efficacy. Secondly, additional mechanistic information and metabolite markers may be identified by using MS-based metabolomics techniques which provide complementary information to NMR. Thirdly, incorporating other omics techniques, such as transcriptomics, may enhance the comprehensiveness and completeness of the research framework, thereby enriching our understanding of the underlying mechanisms.

## 5. Conclusions

To assess the potential anti-aging effects of hemp seed oil in rats with D-gal-induced aging, we employed a comprehensive approach that integrated NMR-based metabolomics with 16S rRNA gene sequencing. Through our study, we discovered that hemp seed oil exhibited a remarkable ability to mitigate age-related metabolic disruptions and restore equilibrium to the intestinal microbiota, thereby improving the aging process in rats. These findings contributed significant mechanistic insights into the underlying anti-aging activity of hemp seed oil.

## Figures and Tables

**Table 1:** Potential biomarkers related to aging in rats.

Metabolite	Chemical Shift (ppm) ^a^	VIP ^b^	FC ^c^	Model ^d^	Hemp Seed Oil ^e^
**Serum**					
VLDL/LDL	0.87 (m), 1.28 (m)	2.91	0.83	↓ *	↑ *
Alanine	1.48 (d)	2.76	1.37	↑ *	↓ *
Glutamine	2.14 (m), 2.45 (m)	1.25	1.84	↑ *	-
Choline	3.20 (s)	2.87	0.82	↓ *	-
Glycine	3.55 (s)	2.71	1.15	↑ *	↓ *
Betaine	3.27 (s), 3.90 (s)	5.24	1.26	↑ *	↓ *
Phenylalanine	3.98 (dd)	1.95	1.61	↑ *	-
β-glucose	4.65 (d)	2.71	1.47	↑ *	-
**Urine**					
Isoleucine	0.94 (t)	1.08	0.61	↓ *	↑ *
Acetate	1.92 (s)	2.73	0.12	↓ *	↑ *
*N*-Acetyl glycoprotein	2.05 (s)	2.42	2.24	↑ *	↓ *
Acetone	2.22 (s)	0.79	0.79	↓ *	↑ *
Acetoacetate	2.30 (s)	0.77	0.70	↓ *	↑ *
Pyruvate	2.38 (s)	1.23	0.57	↓ *	↑ *
α-Ketoglutarate	2.44 (t), 3.01 (t)	1.74	0.54	↓ *	↑ *
Dimethylglycine	2.93 (s)	1.22	0.30	↓ *	↑ *
Cysteine	3.12 (m)	1.47	0.40	↓ *	-
Sarcosine	3.60 (s)	2.45	0.60	↓ *	↑ *
Creatinine	4.05 (s)	2.49	0.45	↓ *	-
Hippurate	7.55 (t), 7.64 (t), 7.84 (d)	2.88	0.46	↓ *	-
Tryptophan	7.33 (s)	0.77	0.59	↓ *	↑ *

^a^ Letters in parentheses indicate the peak multiplicities: s, singlet; d, doublet; dd, doublet of doublet; t, triplet; m, multiplet. ^b^ VIP was obtained from OPLS-DA models (Figure 3B and Figure 4B). ^c^ Fold change (FC) was calculated as the ratio of the mean metabolite levels between model and control groups. FC with a value > 1 indicates a relatively higher concentration while a value < 1 means a relatively lower concentration present in model group as compared to those in the controls. ^d^ Compared to the control group: ↑ indicates a relative increase in signal, while ↓ indicates relative a decrease in signal. ^e^ Compared to the model group: ↑ indicates a relative increase in signal, while ↓ indicates relative a decrease in signal. * Represents *p* < 0.05, whereas - denotes no statistically significant difference.

## Data Availability

All data are included in the manuscript.
